# Human leptospirosis in The Federated States of Micronesia: a hospital-based febrile illness survey

**DOI:** 10.1186/1471-2334-14-186

**Published:** 2014-04-07

**Authors:** Susannah Colt, Boris I Pavlin, Jacob L Kool, Eliaser Johnson, Judith P McCool, Alistair J Woodward

**Affiliations:** 1 The Institute for Immunology and Informatics, Department of Cell and Molecular Biology, University of Rhode Island, Providence, RI, USA; 2 Office of the WHO Representative in Papua New Guinea, World Health Organization, Port Moresby, Papua New Guinea; 3 Division of Pacific Technical Support, World Health Organization, Suva, Fiji; 4 Pohnpei Department of Health Services, Kolonia, Pohnpei State, Federated States of Micronesia; 5 School of Population Health, University of Auckland, Auckland, New Zealand

**Keywords:** Leptospirosis, Pohnpei, Federated States of Micronesia, Serosurvey

## Abstract

**Background:**

Human leptospirosis is an emerging infectious disease of global significance, and is endemic to several countries in the Pacific. Zoonotic transmission dynamics combined with diagnostic challenges lead to difficulties in prevention and identification of cases. The Federated States of Micronesia (FSM) lacks surveillance data for human leptospirosis. This hospital-based serologic survey sought to estimate the burden of leptospirosis, collect information relating to associated factors, and assess the leptospirosis point-of-care rapid diagnostic test (RDT) commonly used in FSM.

**Methods:**

A four-month hospital-based survey was conducted in Pohnpei State, FSM in 2011. Patients with undifferentiated fevers presenting to hospital were referred for enrolment by physicians. Consenting participants provided paired blood specimens 10–30 days apart, and responded to interview questions regarding demographics, clinical symptoms, exposure to animals, and environmental exposure. Blood samples were subjected to immunochromatographic RDT and confirmed by microscopic agglutination test (MAT).

**Results:**

Of 54 participants tested by MAT, 20.4% (95% confidence interval [CI] 10.1–30.6%) showed serologic evidence of acute infection. Occupation student (odds ratio [OR], 17.5; 95% CI: 1.9–161.1) and recreational gardening (OR, 8.6; 95% CI: 1.0-73.8), identified by univariate logistic regression, were associated with infection. The local rapid diagnostic test (RDT) performed with a sensitivity of 69.2 (42.3-89.3 CI) and specificity of 90.0 (81.6-95.6 CI) compared to MAT.

**Conclusions:**

This study demonstrated a high burden of leptospirosis in Pohnpei. Further work is warranted to identify additional risk factors and opportunities to control leptospirosis in Pohnpei and other Pacific settings.

## Background

Human leptospirosis is a widespread zoonotic disease caused by bacteria of the genus *Leptospira*[1]. *Leptospira* are spirochetes with over 200 pathogenic serovars identified worldwide [2]. The global burden of this disease is unknown due to underreporting, misdiagnosis, and lack of confirmatory diagnostic resources [3]. While cases can remain asymptomatic, infection often induces an acute febrile illness with a broad spectrum of symptoms of variable morbidity ranging from mild to fatal. Presentation is described in four clinical categories including a mild influenza-like illness; jaundice, renal failure, haemorrhages and cardiac arrhythmias, termed Weil syndrome; meningitis or meningoencephalitis; and pulmonary haemorrhage with respiratory failure [4]. Untreated, Leptospirosis can result in chronic morbidities and in some cases death. Rough estimates measure case fatality between <5 to 30% worldwide [2]. Human transmission occurs from direct contact with carrier animals or via indirect contact with pathogenic leptospires present in the environment [5]. *Leptospira* carrier animals, including cattle, pigs, dogs, and rodents, excrete high concentrations of the bacteria in urine, contaminating fresh water and soil [6]. Areas most affected are humid tropical or subtropical climates where populations live in close proximity with feral or domestic animals carrying the disease [7]. Leptospirosis is considered an emerging infectious disease of significant concern in tropical developing regions including Pacific Island populations [8].

While published data finds endemic conditions in many Pacific Island countries, it is likely that the true extent is underestimated [9]. Rates in hyperendemic Pacific Island countries including French Polynesia and New Caledonia are much greater than those reported in the Federated States of Micronesia (FSM) [10]. A survey conducted from 2003–2005 found 69 confirmed cases from 263 suspected cases from seven Western Pacific Islands and recommended further investigation, specifically in FSM [11]. Human leptospirosis in FSM was first described in Kosrae State in 1990 (D. Upson, unpublished observations) and then in Pohnpei State in 1991 [12] and 1993 [13], but there has been limited surveillance to date. An animal study conducted from 1995–1996 isolated *Leptospira* from dogs, pigs, and rodents throughout FSM, including Pohnpei State [14]. In 2010, an outbreak investigation in Chuuk State, FSM revealed 2 confirmed cases of leptospirosis (involving different serovars, suggesting more than one independent introduction from animal reservoirs) from a sample of 10 febrile patients [15]. A significant barrier to surveillance is FSM’s lack of laboratory facilities able to produce confirmatory diagnoses. In Pohnpei, the standard of care for hospital patients suspected of leptospirosis is precautionary treatment with antibiotics, although some patients may be given an acute phase rapid leptospirosis titre test if available. For confirmatory analysis, suspected case samples must be shipped overseas.

Risk factors for transmission present in FSM include: a humid tropical climate; close association between animals and humans; horticultural or pastoral livelihoods; slaughter or food preparation of carrier animals; and recreational activities like swimming, hiking, and children’s outdoor play. Pohnpei, FSM experiences high annual rainfall contributing to a potentially endemic environment. Compounding these factors, climate change estimates predict that these conditions will worsen with increasing temperatures and annual rainfall [16].

This study sought to investigate the presence of human leptospirosis in Pohnpei State. Our primary objective was to quantify cases of leptospirosis amongst patients presenting to the health services with undifferentiated fevers and collect information relating to risk factors associated with contracting leptospirosis. A secondary objective of the study was to determine the sensitivity and specificity of the leptospirosis point-of-care rapid diagnostic test used in Pohnpei, when compared with the reference standard microscopic agglutination test (MAT) results.

## Methods

A hospital-based serosurvey was conducted in Pohnpei State, FSM from June-September 2011. Pohnpei State, with a population of roughly 35,000, is served by two main hospitals and several small dispensary clinics. Eligible participants included individuals older than 10 years who presented to either of the hospitals on the island (Pohnpei State Hospital and the private Genesis Hospital) with reported fever or measured temperature ≥ 38 C/100.4 F, and two or more of the following symptoms: headache, fatigue, myalgia, chills, conjunctival suffusion, anuria or oliguria, cough, jaundice, haemorrhages, vomiting, diarrhoea, meningeal irritation, cardiac arrhythmias, or skin rash. Exclusion criteria included diagnosed upper respiratory illness, pneumonia, or influenza. Confirmed diagnosis of dengue would have been considered exclusionary, but dengue is not routinely tested in Pohnpei. Patients were referred to the study by hospital physicians. Written consent was provided by the patient or family member.

Enrolled participants provided paired blood specimens and responded to interview questions. A primary blood specimen was collected at enrolment, and a secondary blood specimen was collected during a follow-up study visit, between 10–30 days following the date of symptoms onset. Collected blood specimens were subject to a rapid leptospirosis titre test (SD BIOLINE Leptospira IgG/IgM test device, Korea) performed at the Pohnpei State Hospital laboratory. Portions of all collected blood serum specimens were saved and frozen for confirmatory MAT, conducted at the WHO/FAO/OIE Collaborating Centre for Reference and Research on Leptospirosis in Queensland, Australia. A positive MAT result was defined as a titre of 1:400 or greater on a single specimen or a four-fold increase between acute and convalescent specimens [8].

Information including demographics, clinical symptoms, exposure to animals, and environmental exposure was collected during a face-to-face participant interview. If participants did not speak English, a translator assisted with the interview. Participants were compensated for time and cooperation.

Statistical analysis was carried out using IBM® SPSS® Statistics 20. Odds ratios (OR) and 95% confidence intervals (CIs) were calculated to measure the association between participant-reported symptoms, demographics, and environmental exposures and leptospirosis cases confirmed by MAT. Logistic regression was used to compare risk factor categories to MAT outcome. Statistical significance was determined using Fisher’s exact test for binomial analyses and Wald’s test for multinomial analyses. Ethical approval was granted by The University of Auckland Ethics Committee and by the Pohnpei State Department of Health.

## Results

During the study period, 66 participants were enrolled out of 69 eligible hospital patients. One patient declined to participate. Two suspected cases died prior to enrolment. On average, patients presented to hospital 3 days (2.12-3.85 CI) following the onset of symptoms. Of those enrolled, 54 were tested for leptospirosis by MAT (Table 1). Some enrolled patients were not tested by MAT if they left the hospital prior to serum collection and could not be contacted by the researcher. Based on MAT results, 20.4% (10.1-30.6% CI) of screened participants were confirmed cases of leptospirosis. The 11 confirmed cases were associated with serovars Copenhageni, Ballum, and LT751-Pohnpei based on a 20 serovar MAT panel (Table 2). A total of 12 distinct serovars were reactive (titre >1:50) in 13 participant serum samples, yet some of these titres measured below diagnostic thresholds. While the MAT serovar panel indicates reactivity, isolation of serovar-specific leptospires is required for definitive typing [17]. Compared to the reference standard MAT, the RDT performed with a sensitivity of 69.2 (42.3-89.3 CI) and specificity of 90.0 (81.6-95.6 CI) on single serum specimens (Table 3). The RDT positive predictive value (PPV) for this sample was 56.3 (32.4-78.2 CI) and the negative predictive value was 94.0 (86.7-98.1 CI).

**Table 1:** Recruitment and enrolment

**Participants**	**Total**
Patients recruited	67
Participants enrolled	66
Participants receiving rapid diagnostic test	62
Both acute and convalescent	42
Acute only	18
Convalescent only	2
Participants receiving MAT	54
Both acute and convalescent	29
Acute only	13
Convalescent only	12

**Table 2:** Serovars detected from MAT

**Serovar**	**Participants with titre >50**	**Confirmed Cases**
Pomona	1	
Sejroe		
Tarassovi		
Grippotyphosa		
Celledoni	5	
Copenhageni	10	5
Australis	4	
Pyrogenes		
Canicola	3	
Hebdomadis	2	
Mini		
Sarmin		
Autumnalis	2	
Cynopteri	4	
Ballum	2	1
Bataviae		
Djasiman	2	
Javanica		
Panama	1	
Shermani		
LT751 (Pohnpei*)	12	5

*Isolated from rodents and pigs during a Pohnpei survey in February 1996. Pohnpei is the proposed name for the LT751 serovar.

**Table 3:** Accuracy of leptospirosis rapid diagnostic test (RDT)

**MAT titre >400**		**Agreement**	**%**	**(95% CI)**
Positive	13	9	69.2	42.3–89.3
Negative	70	63	90.0	81.6–95.6

Amongst those who were screened, occupation was associated with leptospirosis (Table 4). Those in the student occupation group, all under the age of 24 years and enrolled in education levels ranging from primary to tertiary, OR = 17.5 (1.9-161.1 CI, P = 0.012), were more frequently associated with leptospirosis compared to farming and other occupations. Participants who regularly tended gardens or crops were also more likely to test positive for leptospirosis, OR = 8.6 (1.0-73.8 CI, P = 0.035). Other, less certain associations include the link between leptospirosis and age 10–24 years, OR = 4.0 (1.0-16.2 CI); the presence of a wound on the leg or foot at the time of enrolment, OR = 4.4 (1.1-17.8 CI); swimming or standing in fresh water, OR = 3.8 (0.9-15.2 CI); and using fresh water streams as a source for drinking or bathing, OR = 1.7 (0.4-6.4 CI) and OR = 1.2 (0.3-4.7 CI) respectively. Most clinical symptoms reported by participants at the time of enrolment were not found to be strong indicators for leptospirosis (Table 5). Vomiting was more common amongst cases, OR = 4.8 (1.1-20.9 CI, P = 0.041).

**Table 4:** Univariate analysis of demographics and environmental exposures

**Factors by category**	**Case frequency% (n)**	**Odds ratio (95% CI)**	***P *** **value**
Sex			
Male	27.3 (33)	3.6 (0.7–18.5)	0.170
Age			
10–24	35.0 (20)	4.0 (1.0–16.2)	0.077
Occupation			
Other	3.8 (26)	Reference	
Farming	27.3 (11)	9.4 (0.9–103.3)	0.067
Student	41.2 (17)	17.5 (1.9–161.1)	0.012
Education Level			
Below secondary	20.7 (29)	1.0 (0.3–3.9)	1.000
Water source*^†^			
Drinking from stream	26.3 (19)	1.7 (0.4–6.4)	0.496
Bathing from stream	22.7 (22)	1.2 (0.3–4.7)	1.000
Animals around home*^†^			
Pigs	16.3 (43)	0.3 (0.1–1.3)	0.187
Dogs	19.4 (36)	0.8 (0.2–3.2)	0.730
Rats	20.0 (40)	0.8 (0.2–3.8)	1.000
Environment/behaviour*			
Walk through mud^‡^	26.2 (42)	Undefined	0.096
Wound on legs or feet^†^	40.0 (15)	4.4 (1.1–17.8)	0.056
Swim or stand in fresh water^‡^	35.0 (20)	3.8 (0.9–15.2)	0.081
Tend gardens or crops^‡^	31.2 (32)	8.6 (1.0–73.8)	0.035
Recent pig slaughter^†^	16.7 (6)	0.7 (0.8–7.1)	1.000

*Each exposure is not mutually exclusive.

^†^Based on 53 responses.

^‡^Based on 52 responses.

**Table 5:** Univariate analysis of clinical signs and symptoms

**Symptom**	**Case frequency% (n)**	**Odds ratio (95% CI)**	***P value***
Headache	22.9 (48)	Undefined	0.571
Fatigue	23.9 (46)	Undefined	0.322
Neck pain	27.8 (36)	6.2 (0.7–52.7)	0.082
Myalgia	27.0 (37)	5.6 (0.6–47.7)	0.141
Vomiting	34.8 (23)	4.8 (1.1–20.9)	0.041
Conjunctival suffusion	30.0 (30)	4.5 (0.9–23.4)	0.089
Haemorrhage	50.0 (4)	4.4 (0.6–35.9)	0.187
Light sensitivity	35.3 (17)	3.4 (0.9–13.3)	0.143
Joint pain	27.6 (29)	2.7 (0.6–11.5)	0.308
Anorexia	25.0 (36)	2.5 (0.5–13.1)	0.469
Cardiac arrhythmias	30.0 (20)	2.4 (0.6–9.3)	0.296
Difficulty bending neck	28.0 (25)	2.3 (0.6–9.2)	0.313
Diarrhoea	25.0 (28)	1.8 (0.4–6.9)	0.509
Orbital pain	25.0 (24)	1.6 (0.4–6.1)	0.518
Nausea	22.5 (40)	1.5 (0.3–8.6)	0.711
Melanuria	24.0 (25)	1.5 (0.4–5.5)	0.737
Runny Nose	23.8 (21)	1.4 (0.4–5.2)	0.736
Cough	21.9 (32)	1.2 (0.3–4.7)	1.000
Rash	22.2 (9)	1.1 (0.2–6.3)	1.000
Oliguria	20.7 (29)	1.0 (0.3–3.8)	1.000
Shortness of breath	18.8 (16)	0.8 (0.2–3.7)	1.000
Jaundice	14.3 (7)	0.6 (0.1–5.6)	1.000
Abdominal pain	15.8 (38)	0.4 (0.1–1.5)	0.258
Chills	18.8 (48)	0.3 (0.1–2.4)	0.275
Confusion	7.1 (14)	0.2 (0.0–1.9)	0.251

## Discussion

This study represents the first serosurvey to identify cases of human leptospirosis in The Federated States of Micronesia, and it confirms that the burden of disease due to leptospirosis is high. It was suspected that *Leptospira* infection occurred in Pohnpei State, but very few confirmed cases had been documented by the State Department of Health Services. The gold standard MAT is available in only a few reference laboratories worldwide, rendering cost and clinical timeliness prohibitive. During the four month recruitment period, 11 hospital patients seeking treatment for febrile illness in Pohnpei State tested positive for leptospirosis by MAT. For the purposes of this study, subjects with single specimen titres of 1:400 or higher were considered MAT positive, but in highly endemic settings, a single MAT specimen may not be as specific as a four-fold increase between paired sera specimens. This is a small study, and further investigation is needed to confirm the magnitude of the problem. However, our findings suggest that leptospirosis may be widely distributed throughout Pohnpei State. Of note also, the study suggests that serovar LT751 is present in humans in FSM. This serovar was first identified in rats and pigs in Pohnpei in 1995 [14], was tentatively named “Pohnpei,” and is currently undergoing confirmation by the International Committee on Systematic Bacteriology [18].

Amongst people attending hospital with fever, occupation was linked to *Leptospira* infection, as was the regular tending of gardens or crops. While farming as an occupation was not more common amongst the leptospirosis cases, tending gardens or crops is an activity reported by participants across all occupation categories. Tending crops of banana, taro, and yam are common for Pohnpeian males while females maintain the land and gardens surrounding the home. Although rainfall in Pohnpei is high with an annual accumulation of 187 inches (475 cm) per year, seasonal distribution of leptospirosis cases was not considered in this study due to the low seasonal variability of rainfall in Pohnpei [19]. Interestingly, the presence of pigs, dogs, or rats was not associated with leptospirosis cases, presumably because these animals are found near most Pohnpeian homes. While pig slaughter is customary at cultural feasts, this factor was not linked to cases as few participants reported involvement with recent slaughter. The positive association between being a student and acquiring leptospirosis is intriguing. While specific risk factors amongst students were not further explored, it is possible that certain behaviours characteristic of school-aged persons in Pohnpei (e.g., playing in mud, caring for domestic pigs, and routine travel by foot) may put such persons at increased risk. This could be explored in future studies.

Affordable and effective diagnostics are necessary in developing settings, where leptospirosis burden is highest. Evaluation for the specific RDT locally available to Pohnpei State hospitals had not been performed in FSM and is not widely referenced in the literature. In this study, the RDT performed well against the MAT but not as accurately as the package insert indicates [sensitivity of 96.3 (53/55) and specificity of 95.3 (143/150)] (SD BIOLINE Leptospira IgG/IgM Test Package Insert, unpublished observations). As indicated by the PPV, only 56.3% of serum specimens positive by RDT were found positive by MAT. In addition to RDT performance, the timing of the RDT compared to patient seroconversion must be considered. Given that patients presented to hospital an average of 3 days following symptoms, and the RDT is unreliable before 7 days of symptoms, the RDT should not be utilized as a clinical diagnostic tool in the acute phase. Therefore, clinicians should continue to treat patients based on clinical symptoms in the absence of MAT, reserving the RDT for public health surveillance. As RDT performance is dependent on serovar-specific antibodies within a given geographic setting [20], further evaluation of rapid tests commonly used in the Pacific context is recommended.

The major limiting factor in this study is the number of participants. The study period of four months was sufficient to confirm leptospirosis cases amongst patients presenting to hospital with undifferentiated febrile illness, but the study sample is too small to permit a comprehensive assessment of risk factors, to establish any seasonal pattern, or to fully evaluate the local RDT against the reference standard.

## Conclusions

Further efforts to conduct active leptospirosis surveillance throughout FSM are recommended. As rapid test sensitivity for acute phase samples is not reliable [21], and MAT is not locally available, routine convalescent RDTs of suspected cases will strengthen surveillance. Control efforts must focus on limiting interaction with carrier animals and preventing animal urine contamination of fresh water streams. Strengthening community awareness and education regarding the transmission and risks of leptospirosis is paramount. Additional research is warranted to investigate more closely the frequency of human leptospirosis throughout the other FSM states.

## Abbreviations

FSM: The Federated States of Micronesia; MAT: Microscopic agglutination test; RDT: Rapid diagnostic test.

## Competing interests

The authors declare that they have no competing interests.

## Authors’ contributions

SC, BIP, JLK, JPM, and AJW conceived of the study and took part in study design. SC conducted the fieldwork, collected the data, performed statistical analysis, and drafted the manuscript. EJ contributed to fieldwork logistics and data collection. BIP and JLK contributed to data analysis and results interpretation. All authors were involved in writing of the final manuscript. All authors read and approved the final manuscript.

## Pre-publication history

The pre-publication history for this paper can be accessed here:

http://www.biomedcentral.com/1471-2334/14/186/prepub
