# Correction: Human leptospirosis in The Federated States of Micronesia: a hospital-based febrile illness survey

**DOI:** 10.1186/1471-2334-14-267

**Published:** 2014-06-04

**Authors:** Susannah Colt, Boris I Pavlin, Jacob L Kool, Eliaser Johnson, Judith P McCool, Alistair J Woodward

**Affiliations:** 1

## Correction

The original article [1] was published with incorrect copyright information. The correct statement should read: “© 2014 World Health Organization; licensee BioMed Central Ltd.

This is an open access article distributed under the terms of the Creative Commons Attribution IGO License (http://creativecommons.org/licenses/by/3.0/igo/legalcode), which permits unrestricted use, distribution, and reproduction in any medium, provided the original work is properly cited. In any reproduction of this article there should not be any suggestion that WHO or this article endorse any specific organization or products. The use of the WHO logo is not permitted. This notice should be preserved along with the article's original URL.”

We apologize for any inconvenience.

## Pre-publication history

The pre-publication history for this paper can be accessed here:

http://www.biomedcentral.com/1471-2334/14/267/prepub
